# Colorectal cancer microbiome programs DNA methylation of host cells by affecting methyl donor metabolism

**DOI:** 10.1186/s13073-024-01344-1

**Published:** 2024-06-05

**Authors:** Zhi Liu, Qingqing Zhang, Hong Zhang, Zhongyuan Yi, Huihui Ma, Xiaoyi Wang, Jingjing Wang, Yang Liu, Yi Zheng, Weijia Fang, Ping Huang, Xingyin Liu

**Affiliations:** 1 Department of Pathogen Biology-Microbiology Division, State Key Laboratory of Reproductive Medicine, Key Laboratory of Pathogen of Jiangsu Province, Key Laboratory of Human Functional Genomics of Jiangsu Province, Center of Global Health, Nanjing Medical University, Nanjing, 211166 China; 2 Core Facility Center, The First Affiliated Hospital of Nanjing Medical University, No. 300 Guangzhou Road, Nanjing, 210029 China; 3 The Affiliated Suzhou Hospital of Nanjing Medical University, Suzhou Municipal Hospital, Gusu School, Nanjing Medical University, Suzhou, 215008 China; 4 Department of Medical Oncology, The First Affiliated Hospital, Zhejiang University School of Medicine, Hangzhou, 310003 China; 5 Department of Surgery, The Third Affiliated Hospital, Nanjing Medical University, Nanjing, 211166 China

**Keywords:** Colorectal cancer, DNA methylation, Microbiota, Methionine metabolism

## Abstract

**Background:**

Colorectal cancer (CRC) arises from complex interactions between host and environment, which include the gut and tissue microbiome. It is hypothesized that epigenetic regulation by gut microbiota is a fundamental interface by which commensal microbes dynamically influence intestinal biology. The aim of this study is to explore the interplay between gut and tissue microbiota and host DNA methylation in CRC.

**Methods:**

Metagenomic sequencing of fecal samples was performed on matched CRC patients (*n* = 18) and healthy controls (*n* = 18). Additionally, tissue microbiome was profiled with 16S rRNA gene sequencing on tumor (*n* = 24) and tumor-adjacent normal (*n* = 24) tissues of CRC patients, while host DNA methylation was assessed through whole-genome bisulfite sequencing (WGBS) in a subset of 13 individuals.

**Results:**

Our analysis revealed substantial alterations in the DNA methylome of CRC tissues compared to adjacent normal tissues. An extensive meta-analysis, incorporating publicly available and in-house data, identified significant shifts in microbial-derived methyl donor-related pathways between tumor and adjacent normal tissues. Of note, we observed a pronounced enrichment of microbial-associated CpGs within the promoter regions of genes in adjacent normal tissues, a phenomenon notably absent in tumor tissues. Furthermore, we established consistent and recurring associations between methylation patterns of tumor-related genes and specific bacterial taxa.

**Conclusions:**

This study emphasizes the pivotal role of the gut microbiota and pathogenic bacteria in dynamically shaping DNA methylation patterns, impacting physiological homeostasis, and contributing to CRC tumorigenesis. These findings provide valuable insights into the intricate host-environment interactions in CRC development and offer potential avenues for therapeutic interventions in this disease.

**Supplementary Information:**

The online version contains supplementary material available at 10.1186/s13073-024-01344-1.

## Background

Colorectal cancer (CRC) ranks among the leading causes of cancer-related death worldwide [1]. The development of CRC involves a complex interplay between genetic mutations, epigenetic alterations, and environmental factors [2, 3]. Commensal microbes interact closely with intestinal epithelial cells and serve as a crucial source of environmental stimuli that can profoundly shape host cellular function. Recent progress in sequencing and computational techniques has facilitated the exploration of the intricate role of gut microbiota in the context of colorectal cancer [4–6], and several recent studies have performed meta-analysis of gut microbiota from different CRC cohorts and identified novel microbial biomarkers [7, 8].

DNA methylation is a prominent epigenetic modification primarily occurring at CpG dinucleotides. It plays a pivotal role in a wide range of physiological and pathophysiological processes by influencing protein binding and chromatin structure associated with tumor formation [9, 10]. Genome-wide hypomethylation was one of the first aberrant methylation events reported in CRC and constituted an early event in colorectal carcinogenesis [11, 12]. Disrupted DNA methylation patterns are closely linked to CRC, as they are associated with transcriptional suppression of tumor-suppressor genes and activation of proto-oncogenes. For example, aberrant hypermethylation in promoter regions of key genes such as CDKN2A, MLH1, and APC serves as crucial markers promoting CRC progression [13, 14]. Despite these findings, the underlying mechanism behind altered DNA methylation patterns in tumor tissue remained largely elusive.

DNA methylation is orchestrated by enzymes termed methyltransferases, which utilize S-adenosyl L-methionine (SAM) as an active methyl-group donor. The intricacies of this process extend to methyl donors actively participating in one-carbon metabolism, encompassing the folate and methionine cycles. DNA methylation deficits are postulated to stem from a broad reduction in one-carbon metabolites, the synthesis of which is contingent upon microbial products originating from the gut [15].

A few studies have attempted to resolve the effects of the gut microbiome or specific microbial taxa on DNA methylation in colonic epithelial cells. Exposure to probiotics or pathogenic bacteria contributed to differential DNA modification patterns in fetal and adult epithelial cells, potentially rendering them susceptible to or protected against various diseases [16]. Notably, in a porcine model, differential DNA methylation was observed in the colonic epithelium between two groups of pigs with variations in the total load and community structure of bacteria. Moreover, the postnatal gut microbiome has been implicated in guiding the postnatal epigenetic processes [17]. Iradj et al. provided constitute evidence that the relative abundance of some bacterial taxa within the microbiota in CRC is significantly associated with methylation or demethylation of host genes in CRC cohort and germ-free mice model [18]. Xia et al. conducted fecal microbiota transplant (FMT) experiments to conventionalize germ-free (GF) mice and demonstrated that the reconstructed gut microbiota significantly increased DNA methylation at multiple CpG sites within tumor suppressor genes [19]. In the mouse model, Ihab et al. suggested that microbiota-induced DNA methylation programming is necessary for intestinal homeostasis in vivo [20]. Collectively, these researches highlight an essential link between the gut microbiome and the host-cell methylome. However, the microbiome-methylome axis in CRC has not been systematically investigated.

In this study, we aimed to elucidate the intricate interplay between microbiota and DNA methylation of host cells in colorectal cancer (CRC) tissues and their corresponding adjacent normal tissues. To achieve this, we integrated metagenomic sequencing analysis and whole genome bisulfite sequencing (WGBS) analysis. Through our comprehensive approach, we identified potential protein-coding genes and noncoding RNAs that exhibited epigenetic and differential regulation by specific taxa in both normal and tumor tissues. Our findings suggest that bacteria associated with CRC may have the ability to influence DNA methylation patterns in colonic epithelial cells, thereby exerting control over intestinal homeostasis or driving the development of intestinal tumorigenesis.

## Methods

### Patient recruitment and informed consent

Adults aged 18 years and older identified as candidates for surgical intervention for colorectal cancer were recruited voluntarily from the Third Affiliated Hospital of Nanjing Medical University, located in Nanjing, China. Exclusion criteria encompassed recent administration of chemotherapy, radiation therapy, or antibiotics within the 14 days preceding enrollment, leading to the inclusion of 24 subjects (Additional file 2: Table S1). Tumor specimens, along with adjacent normal tissues situated 2 to 3 cm away from the tumor (referred to as AN), were collected during surgical resection procedures conducted at the Department of General Surgery. The samples were isolated and snap-frozen in liquid nitrogen immediately, and all samples were stored at − 80 °C before use. Feces were collected at the hospital. The feces were collected in a 10-mL sterile container and delivered immediately at low temperatures. The frozen feces were shipped using dry ice overnight to Nanjing Medical University. Once received, fecal samples were divided into three parts of 200 mg and stored at − 80 °C until extraction. All subjects were given written informed consent to participate in the study. The study was approved by the research ethics committee at the Third Affiliated Hospital, Nanjing Medical University (2018-SR-24).

### Tissue bacterial DNA extraction, sequencing, and sequence processing

DNA extraction and library preparation on colon tissue (tumor and normal-adjacent) were performed using a bacterial DNA extraction kit (IndiSpin Pathogen Kit) following the manufacturer’s recommendation, which is designed for the extraction of pathogen nucleic acids (viral RNA and DNA, and bacterial DNA) from animal whole blood, serum, plasma, other body fluids, swabs, washes, and tissue. PCR was performed to produce V4 regions of the 16S rRNA gene using the conserved primers 515F (5′-GTGCCAGCMGCCGCGGTAA-3′) and 806R (5′-GGACTACHVGGGTWTCTAAT-3′), and no template DNA reaction was used as a negative control. Polymerase chain reaction (PCR) products were purified using the GeneJET Gel Extraction Kit (Thermo Scientific, USA). Following the manufacturer’s recommendation, libraries were generated using the Illumina TruSeq DNA PCR-Free Library Preparation Kit (Illumina, USA). A total of 24 tumors and 24 matched adjacent normal tissue microbiome libraries were quantified for sequencing. Then the PCR fragments were sequenced in the Illumina NovaSeq platform (Novogene, China). In addition, to account for external contamination sources, we applied a blank control of microbial DNA-free water that run alongside the biological samples.

Bioinformatics analysis of 16S rRNA gene amplicons was performed by Qiime2 (version 2020.8.0) [21]. Briefly, fastq reads were processed by the dada2 program, and dada2 denoise-paired commands were used to delete the low-quality ones. Dada2 generates unique features that could be compared between different studies. The taxonomy of these features was assigned to the Silva reference database (version 138) [22] classifier with 99% similarity. The taxa with a relative abundance > 0.0001 in more than 10% of samples were retained at each taxonomic level. Determination of alpha and beta diversities was conducted by R packages vegan.

The functional capacity of the gut microbial community was imputed using PICRUSt2 from the original microbial abundance. Predicted functional genes were categorized into MetaCyc Enzyme Consortium (EC) pathways.

Linear discriminant analysis (LDA) effect size (LEfSe) algorithm with an *α* < 0.05 and LDA score > 2 (on a log10 scale) was applied to identify the enriched and significant bacteria and bacterial functions. *P*-values were corrected from 10,000 random permutations.

### Shotgun sequencing for metagenomics

Feces from 18 patients and 18 healthy control samples were subjected to shotgun sequencing. Sequence libraries were generated using NEBNext® Ultra™ DNA Library Prep Kit for Illumina (NEB, USA). The libraries were sequenced on the Illumina Novaseq 6000 platform (insert size 350 bp, read length 150 bp) at the Novogene Bioinformatics Technology Co., Ltd. (Tianjin, China). Raw sequence reads were trimmed using Trimmomatic v0.39 to remove adapters and low-quality regions. Contaminating human reads were removed using Bowtie2 v2.4.2 against GRCh38. The taxonomic composition was profiled using the default parameters of MetaPhlAn3 v3.0.9 [23]. Functional potential profiling of microbial communities was performed by HUMAnN3 [24] using pangenomes annotated with UniRef90 on all species detectable per sample with MetaPhlAn3. Linear discriminant analysis (LDA) effect size (LEfSe) algorithm with an *α* < 0.05 and LDA score > 2 (on a log10 scale) was applied to identify the enriched and significant bacteria and bacterial functions. *P*-values were corrected from 10,000 random permutations.

### Meta-analysis of microbial pathways

Meta-analysis on the pathways was performed on the collection of publicly available metagenomic datasets (Additional file 2: Table S2) and 16S rRNA datasets (Additional file 2: Table S3). The pathway abundance table of metagenomic data was obtained from public datasets provided by Beghini et al. [24]. The pathway and enzymatic functions abundance table of 16S rRNA datasets were generated by processing the raw fastq data downloaded from the NCBI database with the pipeline described above. The meta-analysis was conducted based on the abundance table. Briefly, relative abundances of pathways were arcsine-square-root transformed, and Cohen’s *D* was computed by the escalc function in the R package “metafor” to model random effects. *I*^2^ statistics and FDR were used for quantifying study heterogeneity and assessing their statistical significance (*I*^2^ < 50%, FDR < 0.05).

### WGBS library construction, sequencing

Genomic DNA was purified using TIANamp Genomic DNA kit (TIANGEN, DP304) for further detection. Genome integrity was assessed by 1% agarose gel electrophoresis. The purity of DNA was evaluated using a K5500 spectrophotometer to ensure that the OD260/OD280 ratio of DNA was in the range of 1.8∼2.0. The Qubit® 3.0 Fluorometer (Life Technologies, USA) was used for the quantitation of DNA using the highly sensitive and accurate fluorescence-based Qubit ® quantitation assays. Thirteen pairs of quantified biospecies from the tumor and AN tissues were selected for library construction. One-microgram qualified genomic DNA from each sample was used to construct the library.

Eighty microliters of genomic DNA spiked with unmethylated lambda DNA was fragmented into 300 bp, followed by terminal repairing and A ligation. The DNA bisulfite conversion was performed using EZ DNA Methylation-Gold™ Kit (Zymo Research, Irvine, CA, USA). Then, the KAPA HiFi DNA Polymerase was used to amplify uracil-containing DNA. Next, the concentration of the WGBS library was quantified using a Qubit 3.0 fluorometer (Life Technologies, Carlsbad, CA, USA). The insert size was checked by Agilent 2100 Bioanalyzer, and the concentration of library was assessed by StepOnePlusTM Real-Time PCR system library. The qualified library with a concentration of more than 10mM was used for sequencing. Finally, the WGBS library was sequenced on an Illumina Novaseq 6000 sequencer as paired-end 150-bp reads by Annoroad (Annoroad, Beijing, China).

### WGBS data processing

Program fastp was used to generate sequence quality reports and to trim low-quality bases and the adapter sequences to obtain high-quality reads. Then, the cleaned data for each sample were processed by Bismark software (0.14.5) [25], including alignment to GRCh38.p12, de-duplication, and base-level methylation calling. Detection of differentially methylated loci (DML) and differentially methylated regions (DMR) was applied using an R package (DSS, R version 4.1.1) [26]. CpG loci with a depth greater than 10 × were retained for analysis. Smoothing with a window size of 500 bp was applied to estimate mean methylation levels as recommended by the DSS User Guide. The DMLs were defined as a methylation difference greater than 20% and *q* value < 0.05. Regions with an average methylation difference greater than 20% and *q* value < 0.05 were defined as DMRs.

### Process of TCGA methylation and microbiome data

Illumina Human Methylation 450 Beadchip (450 K array) of TCGA-CRC cohort was downloaded from The Cancer Genome Atlas (TCGA) database (https://tcga-data.nci.nih.gov/tcga/). Forty-five patients with matched methylation data from the tumor and adjacent normal tissues were retained for analysis. R package ChAMP was applied to process the level 3 methylation data. First, data were cleaned and normalized. Then, the differentially methylated CpG sites (DML) and regions (DMR) were identified with default parameters. Enrichment analysis for the differentially methylated genes was performed with R package missMethyl [27] to control the “probe-number bias” for each gene.

The microbial abundance profile (count data) was provided by Poore et al. [28], where they re-examined treatment-naïve whole genome and transcriptome sequencing from the TCGA samples for microbial reads and quantified the microbial abundances.

### Association analysis

We performed a lasso penalized regression to identify associations between individual DML and gut microbial taxa, followed by a stability selection to select robust associations using the methods described by Priya et.al [29]. We implemented a locus-wise model using the methylation level for loci as dependent variables and abundances of microbiome taxa as predictor variables to identify microbial taxa (CLR-transformed) or functions that are correlated with a DML. Briefly, the variable selection was performed by the lasso regression using shrinkage or regularization, picking only a few taxa associated with a host gene methylation. R package “hdi” (version 0.1–7) was used to perform the estimation of confidence intervals and hypothesis testing in high-dimensional and sparse settings. Moreover, the multiple hypothesis testing was corrected with Benjamini-Hochberg (FDR) method. To account for other factors that can influence host gene methylation or microbial composition, covariates in the predictor matrix for gender (male or female), age, and disease subtype of CRC (COAD or READ) were included in the model.

Next, the R package “stabs” (version 0.6–3) was used to perform stability selection. Finally, an intersection was performed between associations identified by the lasso model described above (FDR < 0.1) and associations identified by stability selection here. We filtered out any significant and stability-selected methylation-gender, methylation-age, and methylation–disease subtype associations from the results to retain significant and stability-selected host methylation–microbe associations at FDR < 0.1.

## Results

### Altered bacteria and bacterial methyl donor-related functions in gut microbiome of CRC

**Fig. 1:**
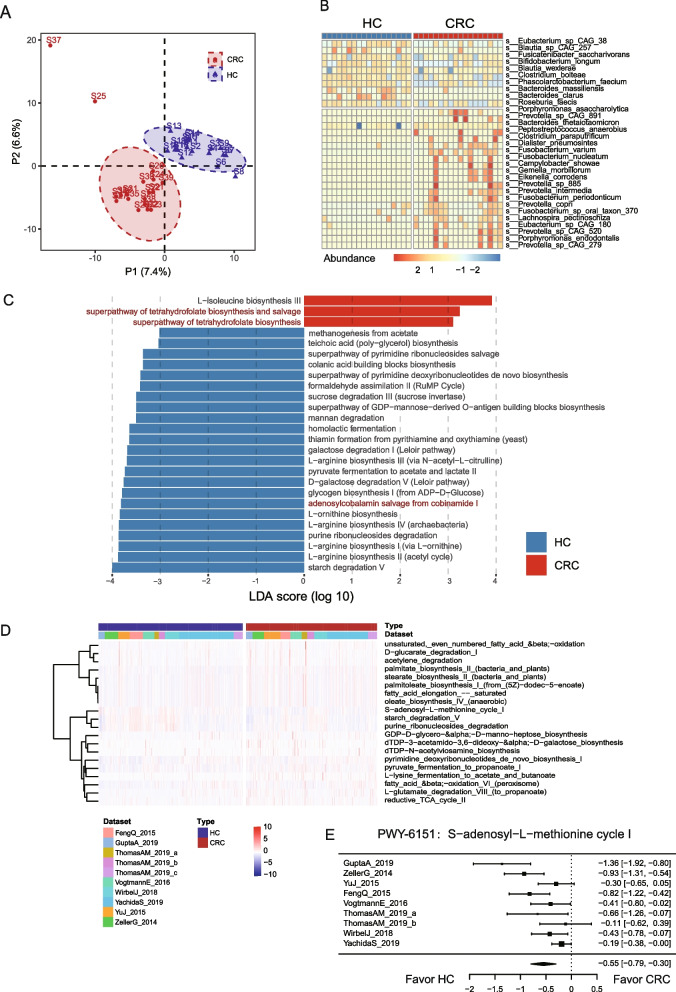
The fecal microbiome in CRC patients and healthy controls. **A** β diversity (based on Bray–Curtis distances) evaluated by PLS-DA analysis. **B** Differential bacterial species between CRC and healthy controls (HC). **C** Differential bacterial pathways between CRC and HCs (pathways with LDA score > 3 are presented). Pathways related to methionine metabolism are highlighted in red. **D** The top 20 differential pathways identified by a meta-analysis based on the fecal microbiome of CRC and HCs derived from 10 public cohorts. **E** Forest plot of the S-adenosyl-L-methionine cycle I pathway

Metagenomic sequencing was performed for fecal samples from 18 CRC patients and 18 healthy controls (HC) (Additional file 1: Fig. S1A). Alpha diversity indexes indicating community richness, diversity, and evenness were assessed via Richness, Shannon, Simpson, Pielou, Chao, and ACE indexes. No statistically significant changes in α diversity were observed in the comparison between CRC and HCs (Additional file 1: Fig. S2). Partial Least Squares Discriminant Analysis (PLS-DA) analyses were performed to evaluate β diversity. Overall fecal microbiome community of CRC patients was different from that of HCs as indicated by PLS-DA analysis (Fig. 1A). The comparison of relative species abundance between HCs and CRC patients revealed the enrichment of several previously reported species associated with CRC in CRC fecal samples, such as *Fusobacterium nucleatum* (LDA = 2.5, adjusted *p* = 0.044) and *Bacteroides thetaiotaomicron* patients (LDA = 4.2, adjusted *p* = 0.012) (Fig. 1B, Additional file 2: Table S4). Furthermore, the investigation of microbial functions associated with CRC identified 35 bacterial pathways that met our criteria (LDA > 2, *α* < 0.05) (Fig. 1C, Additional file 2: Table S5). Notably, multiple pathways related to methionine metabolism were prominently observed (Fig. 1C, highlighted in red). For instance, the COBALSYN − PWY (adenosylcobalamin salvage from cobinamide I, LDA = 3.8, adjusted *p* = 0.004) exhibits enrichment in the HCs. Adenosylcobalamin, also recognized as coenzyme B12, acts as a coenzyme, enabling the transfer of methyl groups during the synthesis of methionine. Nevertheless, the pathways associated with tetrahydrofolate biosynthesis, including both the super pathway of tetrahydrofolate biosynthesis (LDA = 3.1, adjusted *p* = 0.007) and the super pathway of tetrahydrofolate biosynthesis and salvage (LDA = 3.2, adjusted *p* = 0.008), exhibited notable over-representation in colorectal cancer (CRC) samples. Tetrahydrofolate, along with its derivatives collectively termed folates, serves as indispensable cofactors in one-carbon metabolism. These molecular entities play a pivotal role in transporting and donating C1-units, which are crucial for the synthesis of methionine and various other metabolites.

To further validate the findings of the dysregulation of methionine metabolism in CRC, we conducted a meta-analysis using fecal metagenomic datasets from 10 public CRC cohorts [30] (Additional file 1: Fig. S1B). The top 20 differential represented pathways between CRC and normal biopsies are presented in Fig. 1D. Consistent with our cohort analysis, we observed a significant alteration in methionine metabolism-related pathways. Particularly, the S-adenosyl-L-methionine cycle I pathway, where the S-adenosyl-L-homocysteine is recycled back to SAM, was reduced in CRC fecal samples across the cohorts (Fig. 1E). These findings further strengthen the evidence for dysregulated methyl donor-related pathways in CRC compared with HCs.

### Altered methionine metabolism in tumor tissue microbiome

**Fig. 2:**
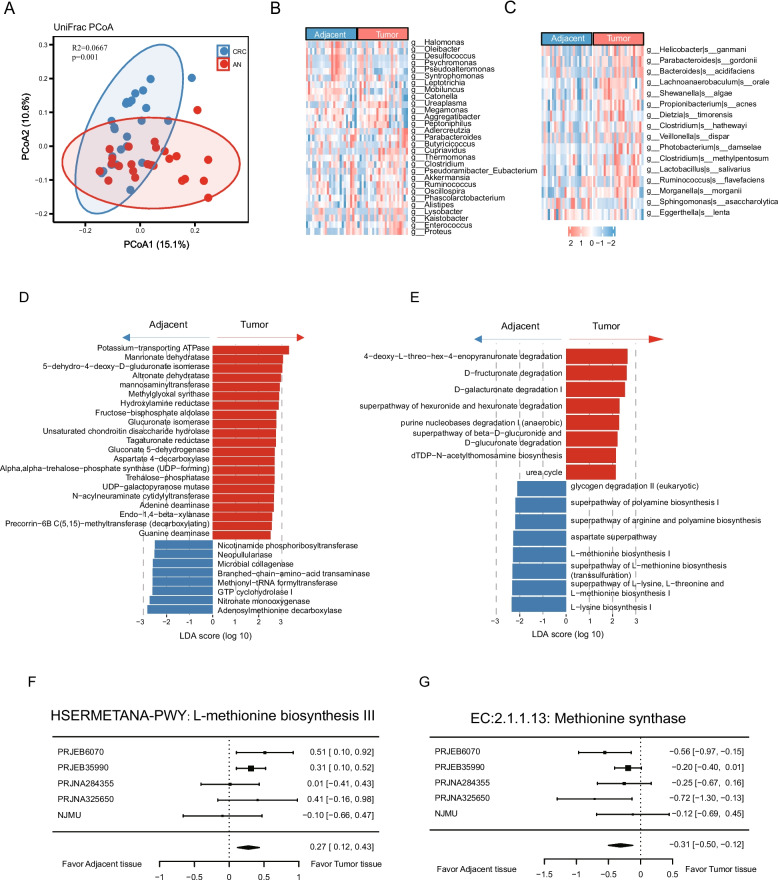
The overview of the microbiome in tumor and adjacent normal tissues. **A** β diversity (based on BrayCurtis distances) evaluated by PCoA analysis. **B**, **C** The differential taxa between CRC and AN tissues at genus (**B**) and species (**C**) level. **D**, **E** The differential microbial enzymatic functions (**D**) and MetaCyc pathways (pathways with LDA score > 2.5 are presented) (**E**) between tumor and AN tissues. **F**, **G** Forest plot of the associations between the relative abundance of L-methionine biosynthesis III pathway (**F**) and Methionine synthase function (**G**)

To explore the microenvironment regarding microbiome in tumor tissues directly, we conducted microbiome profiling using 16S rRNA gene amplicon sequencing in 24 pairs of tumor and adjacent normal (AN) biospecies (Additional file 1: Fig. S1C). There are 1008 ASVs, which are annotated to 171 bacterial genera and 146 species, passed our criteria (> 0.0001 in more than 10% of samples). The paired analysis revealed differences in microbial structure and composition between tumor and AN tissues of CRC patients (Fig. 2A–C). Though the alpha diversity remained comparable between tumor and AN tissues (Additional file 1: Fig. S3), significant dissimilarities were observed in β-diversity (Fig. 2A) as well as microbial abundance at multiple taxonomic levels (Fig. 2B, C). Bacteria that were reported to be associated with CRC, such as *Parabacteroides* (LDA = 3.7, adjusted *p* = 0.030), *Alistipes* (LDA = 3.0, adjusted *p* = 0.002), and *Ruminococcus* (LDA = 3.1, adjusted *p* = 0.039), were significantly enriched genera in tumor tissues (Additional file 2: Table S6). Furthermore, *Clostridium hathewayi* (LDA = 2.7, adjusted *p* = 0.049), a newly recognized CRC pathogen [31], showed a significant over-representation in tumor tissues.

To further investigate the functional implications of the altered microbiota in CRC, we utilized PICRUSt2 to predict MetaCyc pathways and enzymatic functions of the microbiome. Specifically, we discerned notable significant enrichment of functions and pathways associated with methionine metabolism (Additional file 2: Table S7-8). For instance, “Adenosylmethionine decarboxylase” (LDA = 2.8, adjusted *p* = 0.010) and pathways related to L-methionine biosynthesis (L-methionine biosynthesis I: LDA = 2.3, adjusted *p* = 0.047; superpathway of L-methionine biosynthesis: LDA = 2.4, adjusted *p* = 0.040; superpathway of L-lysine, L-threonine, and L-methionine biosynthesis I: LDA = 2.3, adjusted *p* = 0.049) were prominently enriched in the AN tissues, as depicted in Fig. 2D, E. Adenosylmethionine decarboxylase catalyzes the conversion of SAM to S-adenosyl methioninamine, playing a pivotal role in the methionine salvage cycle. In agreement with the analysis of gut microbiota, these findings further highlight the potential involvement of methionine metabolism in maintaining normal DNA methylation patterns in colonic tissues.

Next, to strengthen our findings, we performed a meta-analysis by incorporating our data with four additional 16S rRNA sequencing datasets from CRC and matched AN tissues (Additional file 1: Fig. S1D). The analysis was based on the predicted MetaCyc pathways and Enzyme Commission (EC) classifications. We noted an enrichment of the L-methionine biosynthesis III pathway in the tumor samples, whereas the methionine synthase displayed enrichment in the adjacent normal samples, as illustrated in Fig. 2F, G. These findings underscore a substantial alteration in methionine metabolism between the tumor and adjacent normal tissues.

### The association between fecal and tissue microbiome in CRC

**Fig. 3:**
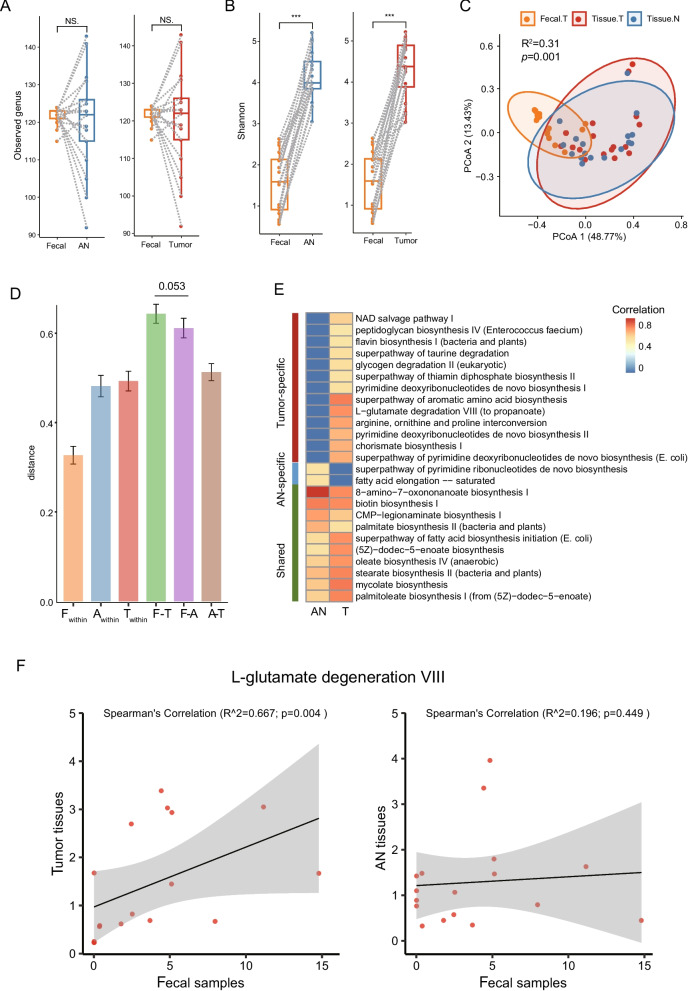
The relationship between microbiome derived from feces and tissues in CRC. **A**, **B** Alpha diversity comparison between feces and tumor/AN tissues from CRC patients. **C**, **D** Beta diversity calculated by PCoA of Bray–Curtis distance among microbiome of feces, tumor, and AN tissues. F, fecal; A, AN; T, tumor. **E** Positively correlated bacterial pathways between fecal microbiome and tumor or AN tissue microbiome. **F** Example pathways of the tumor-specific correlation

A pivotal debate within microbiome research concerns the correlation between gut microbiota composition in feces and that in tissues [32, 33]. We investigated this relationship by examining the association between the fecal microbiome of colorectal cancer (CRC) patients and the tissue microbiome derived from both tumor and AN tissues (Additional file 1: Fig. S1E). At the genus level, the richness of the fecal microbiome did not exhibit any discernible association with that of the tissue microbiome for either tumor or AN tissues (Fig. 3A). However, the Shannon index, which measures both the richness and evenness of the microbiome was significantly higher in tissue samples (Fig. 3B). Beta diversity analysis revealed that the microbiome of fecal samples is distinct from that of tissue samples (Fig. 3C), and the distance from fecal microbiome to tumor microbiome is marginally significantly higher than that to AN microbiome (Fig. 3D). Therefore, these data seem to indicate that microbiota analysis in feces can be considered only a partial representation of the colorectal tissue microbiota.

A taxa-wise correlation analysis between fecal and tissue microbiome revealed few significant associations (Additional file 2: Table S9). For example, the abundance of *Eikenella* is significantly correlated between fecal and tissue. *Eikenella* is a common inhabitant of the oral cavity and the intestinal and genital tracts, and a part of mucosal microbiota. It is considered to be an opportunistic pathogen leading to various infections. Next, we further assessed the congruence in microbial functions between the fecal and tissue microbiomes. Remarkably, we observed positive correlations in the abundance of 23 pathways between the fecal microbiome and tumor tissue microbiome. Likewise, 12 pathways displayed positive correlations between the fecal microbiome and AN tissue microbiome. These associations encompassed 13 pathways specific to tumor tissues, 12 shared pathways between both tumor and AN tissues, and 2 pathways exclusive to tumor tissues (Fig. 3E). This intriguing finding suggests that the functional attributes of fecal bacteria might partially reflect those of the tumor tissue. For instance, the L-glutamate degradation VIII pathway in fecal samples exhibited a robust correlation with the same pathway in tumor tissues, while this correlation was not observed in AN tissues (Fig. 3F). Notably, the meta-analysis conducted using publicly available fecal microbiome data indicated a significant increase in the L-glutamate degradation VIII pathway among tumor samples (Fig. 1D). This line of evidence suggests that specific functions within the fecal microbiome may indeed mirror those within CRC tumor tissues. This finding aligns with the perspective that insights gained from fecal samples could inform conclusions regarding the metabolic and functional profiles of the intestinal microbiota within the tumor microenvironment [34, 35].

### Distinct DNA methylation profiles in CRC tumor and adjacent normal tissues

**Fig. 4:**
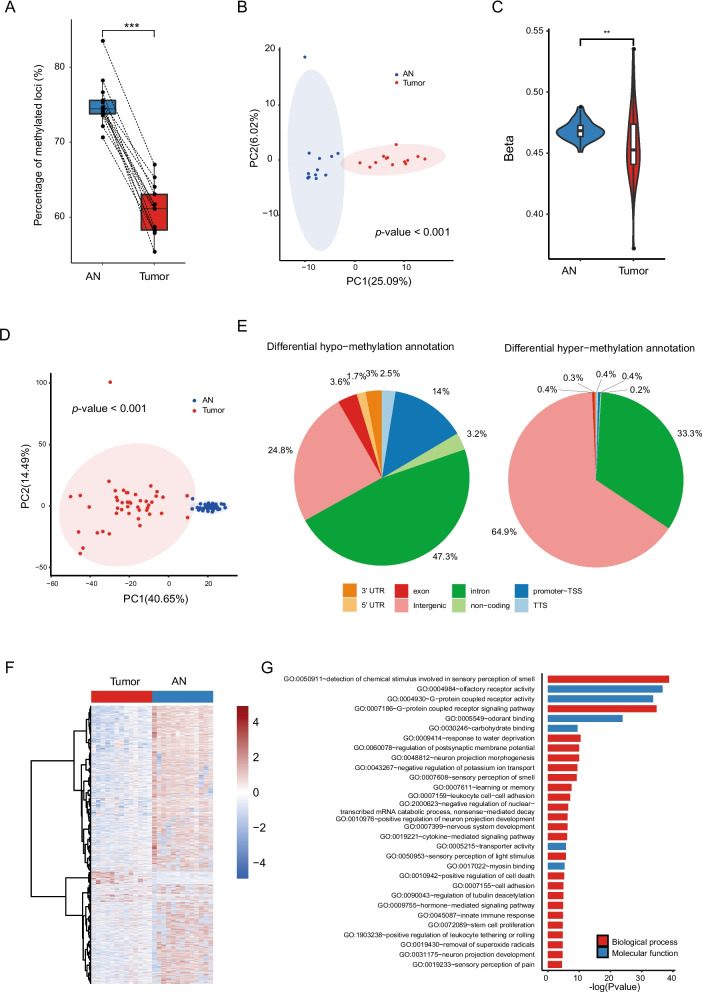
The overview of DNA methylation profile in tumor and adjacent normal (AN) tissues. **A** The pair-wise comparison of the overall methylation level of the CRC tissues and its matched adjacent normal tissues. **B** PCA plot discriminated the tumor and AN tissues. **C** The average methylation level of TCGA CRC tumor and normal biospecies. **D** PCA plot discriminated the tumor and AN tissues in TCGA-CRC cohort. **E** Distribution of significantly hypo-methylated and hyper-methylated regions in a genomic region. **F** The heatmap of differentially methylated loci within promoter regions. **G** Functional enrichment of differentially methylated genes

Genome-wide analysis of DNA methylation was performed using whole-genome bisulfite sequencing (WGBS) on 13 CRC tissue biopsies and their corresponding adjacent normal colonic tissue biopsies (AN) (Additional file 1: Fig. S1C). The CRC tissues exhibited a significantly lower overall methylation level compared to the matched AN tissues, as illustrated in Fig. 4A. Principal component analysis (PCA) analysis showed a sharp difference in the methylation profile between the tumor and AN tissues (Fig. 4B). These findings were further validated through additional analysis using 45 paired tumor and AN tissues from the TCGA-CRC cohort, as shown in Fig. 4C, D.

To comprehensively investigate the genome-wide differential methylation patterns, we identified differentially methylated loci (DML) and regions (DMR). These regions were classified as either hypermethylated or hypomethylated based on an absolute average methylation difference (delta) of > 0.2 between CRC and AN tissues, representing a 20% change in methylation levels. A total of 210,171 hypomethylated regions and 1210 hypermethylated regions in CRC were identified. Notably, the promoter regions were highly hypomethylated, as demonstrated in Fig. 4E and F. Moreover, functional enrichment analysis revealed that the differentially methylated genes were enriched in functions of G-protein coupled receptor signaling pathway and ion transport (Fig. 4G), consistent with the analysis based on TCGA-CRC cohort data (Additional file 1: Fig. S4A).

### Microbial-promoter methylation association was disturbed in the tumors tissues

**Fig. 5:**
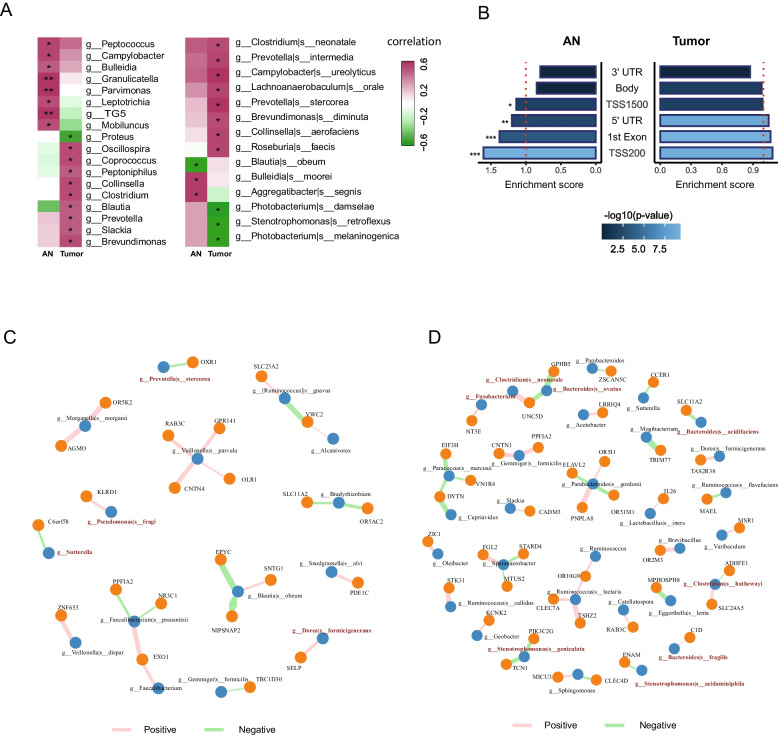
Interactions between intra-tissue bacteria and host DNA methylation. **A** The correlation between bacterial abundance at the genus (left panel) and species (right panel) level with the total DNA methylation level in AN and tumor tissues. **B** The enrichment of bacteria-associated methylation loci in different genomic regions. **C**, **D** The associations between bacteria abundance and promoter CpG loci in the AN (**C**) and tumor (**D**) tissues of our clinical samples

To gain insight into the interplay between the microbiota and DNA methylation in colorectal cancer (CRC) tissues, we examined the association between microbial abundance and host methylation levels. Firstly, we performed a comprehensive analysis that involved correlating the abundance of bacterial taxa at both the genus and species levels with the overall methylation levels in both the host tumor and adjacent normal (AN) tissues. The results of this analysis, as visually represented in Fig. 5A, clearly demonstrate that the abundance of bacteria genera and species associated with global CpG methylation levels exhibited notable distinctions between tumor and AN tissues. This intriguing finding suggests that the microbial composition and its influence on DNA methylation may vary significantly between tumor tissues and the AN tissues in the context of colorectal cancer.

Subsequently, we explored associations between individual host CpG loci and bacterial taxa. We employed a lasso penalized regression model to identify specific microbial taxa whose abundance was correlated with the methylation levels of CpG loci across the entire genome (Additional file 1: Fig. S1F). These models were fitted in a loci-wise manner, with the methylation level of each host CpG loci as the dependent variable and the abundances of microbial taxa as predictor variables. To identify robust associations, a stability selection methodology described by Priya et al. was utilized [29]. Consequently, we identified 2212 significant and stability-selected host CpG methylation-bacteria associations in tumor tissues and 1200 such associations in AN tissues of the TCGA-CRC cohort (Additional file 1: Fig. S1G, Fig. S5B-C).

Previous studies have indicated that specific microbial taxa can regulate the promoter methylation of individual host genes, thereby influencing gene expression [19]. Hence, we annotated the microbial-associated CpG loci to genomic regions. Using the total probes as background, we observed an enrichment of microbial-associated CpGs in promoter-associated regions, including the “TSS200,” “TSS1500,” “1st exon,” and “5′ UTR” region, but a depletion in the gene body and 3′ UTR region in the AN tissues. Strikingly, this enrichment was absent in the tumor tissues (Fig. 5B). These findings indicated a disturbance of microbial-promoter methylation regulation in the tumor microenvironment.

### Microbes regulate methylation level of promoter regions

Next, we focused on the association between microbes and promoter CpG loci, uncovering 27 and 41 associations between microbial taxa and CpG loci in the AN and tumor tissues in our cohort, respectively (Fig. 5C, D). Notably, in the AN tissues, we observed multiple associations between the health-beneficial bacteria and gene methylation (Fig. 5C). For instance, *Faecalibacterium prausnitzii*, which is known for its abundance in the healthy human microbiota and depletion in various intestinal disorders, including CRC [36–38], was associated with the methylation of PPFIA2, NR3C1, and EXO1. Particularly, the methylation of the EXO1 promoter showed a positive association with the abundance of both *F. prausnitzii* and genus *Faecalibacterium*. EXO1 is involved in checkpoint progression and several DNA-repair pathways [39], and its deregulation is commonly observed in tumors [40, 41]. Analysis of TCGA data also revealed significantly increased EXO1 expression in colorectal tumors (Additional file 1: Fig. S5). Furthermore, *Blautia obeum*, a potential probiotic known to affect the composition of intestinal microbiota and inhibit the colonization and proliferation of pathogenic bacteria [42, 43], exhibited a strong negative association with multiple methylated loci (chr7:55963861, chr7:55963871and chr7:55963874) within NIPSNAP2 promoter. NIPSNAP2 is a mitochondrial membrane protein acting as a mitophagy receptor, the defective mitophagy has been increasingly associated with various diseases, including CRC [44]. These examples suggested a possible role of certain beneficial bacteria in maintaining normal cell functions through epigenetic regulation of gene expression in normal tissues.

In contrast, in the tumor tissues, we observed a greater number of associations involving potential opportunistic pathogenic bacteria (Fig. 5D). For instance, *Bacteroides fragilis* (*B*. *fragilis*) showed a positive association with C1D methylation, while *Clostridium neonatale* was positively associated with UNC5D promoter methylation. Impressively, *Clostridium hathewayi*, a recently identified pathogenic bacteria in CRC [19, 31], exhibited an association with the promoter methylation of SLC24A5 and ADHFE1.

Noncoding RNAs (ncRNAs) play a crucial role in gene expression regulation and are associated with various biological processes, including tumorigenesis in mammals. Dynamic DNA methylation patterns of ncRNAs have been reported during human embryonic development and disease progression [45–47]. WGBS identified a couple of methylation loci within the promoter of noncoding RNAs. In our analysis, we examined the association between noncoding RNA promoter methylation and tissue microbes, identifying 47 and 51 associations in AN and tumor tissues, respectively (Additional file 1: Fig. S7A-B). Notably, the methylation level of CCAT1, a well-known lncRNA involved in multiple tumors, including CRC [48], exhibited a negative association with *Bacteroides ovatus* in tumor tissues and a positive association with *Veillonella parvula*in AN tissues.

Furthermore, we observed associations between the methylation of different sets of small nucleolar RNAs (snoRNAs) and tissue microbiota in AN and tumor tissues (Additional file 1: Fig. S6A-B). snoRNAs are a group of regulatory RNAs mainly located in the nucleolus and guide the acquisition of 2′-O-methylation and pseudo-uridylation modify on ribosomal RNA (rRNA) and small nuclear RNA (snRNA). One of the two main families of snoRNAs, the C/D snoRNA genes, have been shown to be controlled by genomic imprinting [49]. Emerging evidence has demonstrated the significant roles of snoRNAs in cancer [50, 51]. For example, methylation of C/D snoRNA genes SNORD113-7 and SNORD114-1 showed associations with the abundance of *Prevotella stercorea* and *Morganella morganii* in the AN tissues. In tumor tissues, SNORD114-1 methylation was associated with *Thermomonas fusca* and *Psychrobacter marincola* abundance. Additional associations were observed between *Parabacteroides gordonii* and SNORD113-7, as well as between *Bacteroides ovatus* and SNORD114-23.

Collectively, our results highlight the potential involvement of microbial influences in the regulation of DNA methylation at gene promoter regions as well as noncoding RNAs. Moreover, the differential associations in the AN and tumor tissues implicate a distinct impact of microbial composition on the epigenetic landscape in these two contexts.

### Tumor bacteria recurrently associated with host gene methylation

**Fig. 6:**
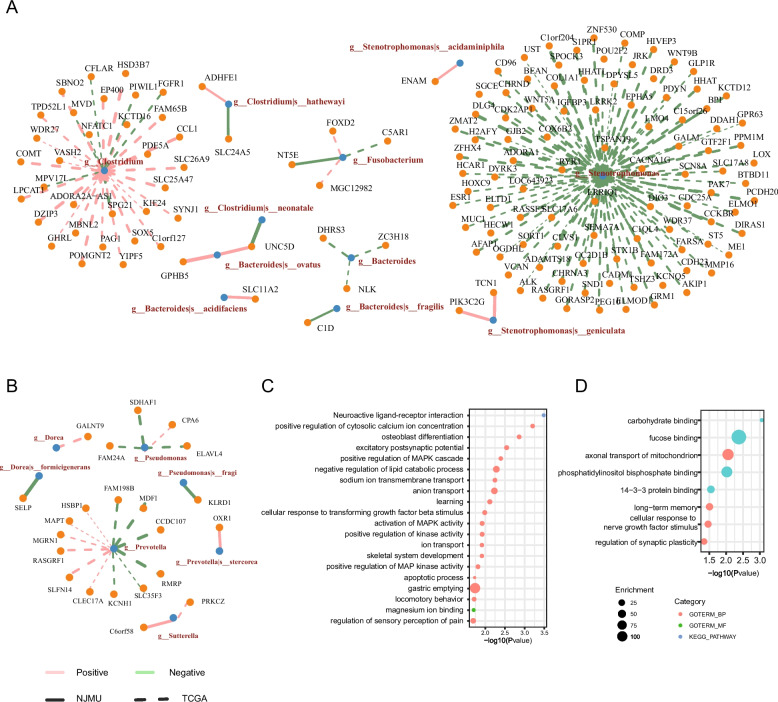
Taxa recurrently associated with host gene methylation. **A**, **B** Taxa commonly associated with host promoter CpG methylation level in the TCGA-CRC cohort and our data (NJMU) in tumor (**A**) and AN (**B**) tissues. **C**, **D** The function enrichment of microbial-associated genes in AN (**C**) and tumor (**D**) tissues

We proceeded to examine the recurrent association between microbes and promoter CpG methylation levels both in the TCGA-CRC cohort and our own data, revealing five genera that exhibited consistent associations with host promoter CpG methylation levels in tumor tissues. These genera include *Fusobacterium*, *Stenotrophomonas*, *Bacteroides*, *Ralstonia*, and *Clostridium* (Fig. 6A). Conversely, the abundance of *Sutterrella*, *Dorea*, *Pseudomonas*, *Corynebacterium*, and *Prevotella* was related to CpG methylation levels in the AN tissues across both datasets (Fig. 6B). Combining the gene methylation associated with these taxa in both TCGA-CRC and our datasets, we observed enrichment of MAPK cascade, ion transport, apoptotic process, and neuroactive ligand-receptor interaction-related functions in microbial-associated genes in tumor tissues (Fig. 6C). Conversely, in the AN tissues, genes were primarily enriched in carbohydrate binding functions (Fig. 6D). Most of the involved species were annotated to be contributed to metabolism of folate and methionine (Additional file 2: Table S10).

*Fusobacterium* and *Clostridium* were well-known taxa associated with CRC progress. Xia et al. reported the role of *Fusobacterium nucleatum* and *Clostridium hathewayi* in driving promoter hypermethylation of tumor suppressor genes (TSG) in colorectal cancer [19]. In our analysis, *Fusobacterium* abundance was positively associated with the methylation level of C5AR1 promoter (cg10224107) in the TCGA-CRC cohort, and NT5E promoter (chr6:85449354) in our clinical samples. C5aR1 is a master regulator in colorectal tumorigenesis through immune modulation. C5a/C5aR1 signaling recruits myeloid-derived suppressor cells (MDSCs) into the inflamed colorectum, impairing CD8^+^T cells and modulating the production of critical cytokines and chemokines, thereby initiating CRC [52]. NT5E (CD73) functions as an inhibitory immune checkpoint molecule, suppressing cellular immune responses [53]. The positive association between *Fusobacterium* abundance and high methylation levels of the C5AR1 promoter is consistent with the potential role of *Fusobacterium nucleatum* in modulating immune responses in tumors, such as creating a proinflammatory microenvironment conducive to colorectal neoplasia progression [54] and enhancing the efficacy of checkpoint inhibitor blockade therapy [55, 56]. Similarly, *Clostridium* abundance was associated with hypermethylation of TSGs, including NFATC1 in TCGA-CRC cohort and UNC5D in our clinical samples. *Stenotrophomonas*, an opportunistic pathogen with increased colonization/infection in cancer patients [57], exhibited extensive positive associations with the hypermethylation of genes, including multiple TSGs in CRC, such as ESR1, RASSF5, DIRAS1, CADM1, ST5, GJB2, FAM172A, and HIVEP3. These findings further support the notion that bacteria in tumor tissue regulate gene expression through modulating methylation of promoter regions.

### Microbial pathways related to host gene methylation

**Fig. 7:**
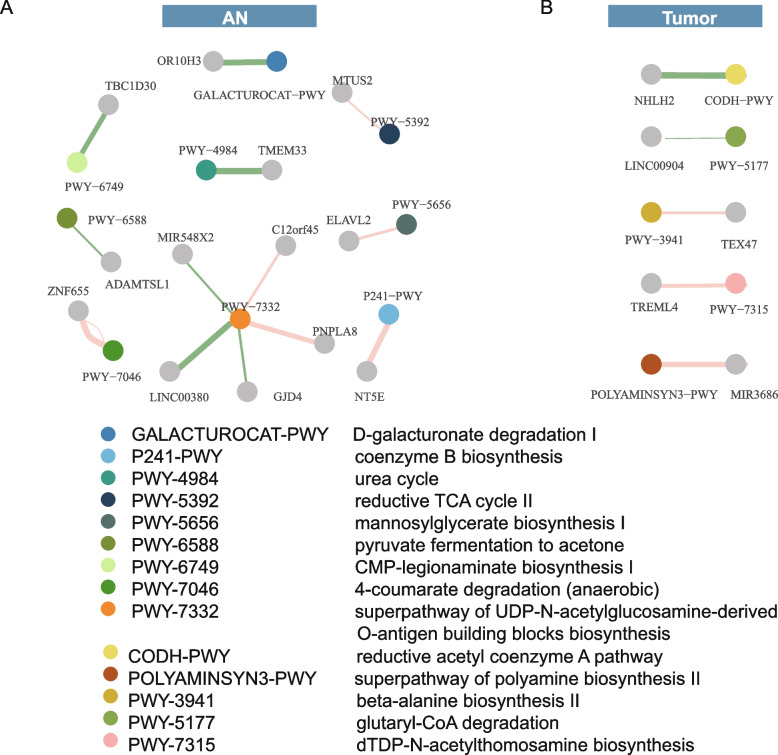
Interactions between microbes and microbial pathways and host DNA methylation. **A**, **B** Microbial pathways associated with host gene methylation level in AN (**A**) and tumor (**B**) tissues

In pursuit of a deeper understanding, we investigated the intricate connection between microbial pathways and the promoter methylation status of host genes (Fig. 7A, B). Notably, a heightened complexity in correlations emerged within adjacent normal (AN) tissues, as compared to tumor tissues. For instance, the “superpathway of UDP-N-acetylglucosamine-derived O-antigen building blocks biosynthesis” exhibited associations with promoter methylation patterns across multiple genes, including C12orf45, GJD4, PNPLA8, MIR548X2, and LINC00380. Of significant interest, C12orf45 serves as a PAQosome cofactor, pivotal in facilitating the assembly of box C/D snoRNP [58]—an event observed to be elevated across various cancer types [59]. Equally noteworthy, the “coenzyme B biosynthesis” pathway demonstrated a positive correlation with NT5E promoter methylation. Importantly, several components of Coenzyme B, specifically B9 (Folate), B12, and B6, play a pivotal role in sustaining the one-carbon transfer cycles, which are integral for providing methyl donors essential for protein and DNA methylation [60].

As demonstrated above, an interrelation between bacterial functions within the fecal and tissue microbiome was established. Subsequently, we extended our analysis to explore associations between the abundance of fecal microbial functions and DNA methylation within tumor tissues. The outcomes unveiled three instances of negative associations between pathway abundance and gene promoter methylation levels (Additional file 2: Table S11). These instances encompassed the L-glutamate degeneration VIII pathway and OR8G5, the L-rhamnose degradation I pathway and C1D, and the superpathway of pyrimidine nucleobases salvage and NPFFR2. Notably, the abundance of the L-glutamate degeneration VIII pathway exhibited a distinctive positive correlation between fecal and tumor tissue microbiome, as depicted in Fig. 3B. This observation is particularly pertinent in light of downstream products resulting from glutamate metabolism, which have been posited to regulate chromatin modifications, including the intricate ten-eleven translocation (TET)-dependent DNA demethylation process [61–63]. Collectively, these findings underscore the multifaceted ways through which the microbiome potentially influences host DNA methylation.

## Discussion

Our present study provides insights into the intricate interplay between methyl donor dynamics within the gut microbiome, host cell DNA methylation, and their implications in both normal and colorectal cancer (CRC) tissues. By conducting an exhaustive analysis of coupled DNA methylation and microbiome profiles from biopsies, encompassing samples from our clinical cohort and the TCGA dataset, we have unveiled noteworthy associations and identified hitherto unknown links between bacteria associated with CRC and promoter methylation patterns of genes implicated in tumor development.

A central discourse in the realm of microbiome research pertains to the interplay between gut microbiota composition within feces and that residing within tissues. In this context, we first embarked on an exploration of this intricate relationship by investigating the association between the fecal microbiome of colorectal cancer (CRC) patients and the microbiome within both tumor and tumor-adjacent normal tissues (AN) tissues to provide clues to guide the judicious selection of specimens for subsequent analyses. Perhaps due to the small size of our sample, limited correlations were observed. For example, the L-glutamate degeneration VIII pathway abundance of the fecal microbiome was specially associated with that of tumor tissue. Glutamine has a versatile role in cell metabolism, participating in tricarboxylic acid (TCA) cycle supplementation and the biosynthesis of nucleotides, glutathione (GSH), as well as L-methionine biosynthesis. Extensive research has been conducted on glutamine metabolism in the intestine, demonstrating its ability to improve intestinal integrity, and the efficacy of glutamine supplementation has been tested in humans and animal models with intestinal diseases [64–66]. These results offered a glimpse into the potential of certain specific functional attributes within fecal bacteria to offer partial insights into the functional characteristics inherent to tumor tissues.

DNA methylation, a dynamic epigenetic modification, involves DNA methyltransferases (DNMTs) catalyzing the addition of a methyl group to cytosine residues (5-hmC) in cytosine-guanine (CG) pairs, resulting in 5-methylcytosine (5-mC). In contrast, TET enzymes facilitate the oxidation of 5-mC to 5-hmC, thereby contributing to a balanced methylation profile in the human genome [67]. Previous investigations have predominantly focused on the influence of microbes on DNA methylation-related enzymes. For example, Xia et al. reported that *F. nucleatum* and *H. hathewayi* activate the expression and activity of DNMT1 and DNMT3A, leading to the suppression of specific TSGs [19]. Another study by Ihab et al. demonstrated the crucial role of TET2/3 in microbiota-induced demethylation using mouse models [20]. Strikingly, our analysis reveals no significant differential expression of DNMTs or TETs in CRC compared to matched normal tissues, as evident from the extensive TCGA database sample (Additional file 1: Fig. S7 and S8). This observation points towards the existence of enzyme-independent mechanisms through which microbes modulate host cell DNA methylation. Intriguingly, our study underscores distinct patterns of methyl donor-related microbial pathways in tumor and normal tissues. This discovery underscores the multifaceted mechanisms through which the microbiome potentially exerts influence over host DNA methylation patterns. To be noticed, we also observed correlations between bacterial pathways related to DNA methylation-related enzymes and gene methylation. The L-glutamate degeneration VIII pathway abundance in fecal samples, which was indicated to be representative of that in the tumor tissues, was linked to promoter methylation of OR8G5. This observation posed the possibility to predict tumor tissue methylation by assessing fecal microbial functions.

To dissect the association between individual taxon and gene methylation, we made two efforts to minimize artificial associations. Firstly, we employed a combination of lasso penalized regression and stability selection to identify only a few taxa that were associated with host gene methylation. Secondly, considering the relatively small sample size of our data, we perform a parallel analysis on both our clinical samples and TCGA-CRC cohort to obtain recurrently identified associations. As a result, several taxa, including *Fusobacterium*, *Stenotrophomonas*, *Bacteroides*, *Ralstonia*, and *Clostridium*, were found to be commonly associated with host promoter CpG methylation levels in tumor tissues, while *Sutterrella*, *Dorea*, *Pseudomonas*, *Corynebacterium*, and *Prevotella* exhibited associations in AN tissues. These findings imply that these bacteria may play essential roles in controlling physical homeostasis and tumorigenesis in both normal and tumor tissues.

Previous studies have revealed the impact of microbiota on the promoter methylation of protein-coding genes [19]. WGBS has provided insights into DNA methylation patterns within the promoters of noncoding RNAs (ncRNAs). ncRNAs are a type of heterogeneous transcript that lack protein-coding potential but possess the ability to regulate the expression of protein-coding genes [68]. Multiple studies have shown alterations in ncRNAs during pathogenic infection. For example, Yang et al. reported differential transcription profiles of long noncoding RNAs in primary human brain microvascular endothelial cells in response to meningitic *Escherichia coli* [69]. We have recently revealed that *Salmonella typhimurium* infection can increase the expression of LINC00152 through histone lactylation, promoting cancer cell invasion and migration [70]. In our analysis, we identified a couple of associations between taxa and ncRNA promoter methylation, including microRNAs (miRNAs), long noncoding RNAs (lncRNAs), and small nucleolar RNAs (snoRNAs). Furthermore, the microbial pathway-based analysis revealed the association between the microbial pathway with the methylation of C12orf45, a PAQosome cofactor that promotes the assembly of box C/D snoRNP [58]. Though the functions of a majority of involving ncRNAs were not fully understood, our observation suggested a prevalent epigenetic connection between the microbiota and noncoding RNAs, particularly snoRNAs.

Due to the lack of transcriptional data from matched samples, we were unable to demonstrate whether differential methylation of promoter regions is being regulated by bacteria and modulating gene expression accordingly. Our current DNA methylation assays were conducted using tissue samples. However, it is crucial to acknowledge that epigenetics plays a pivotal role in bridging the gap between genomics and cellular phenotype. Presently, single-cell epigenome sequencing, coupled with single-cell transcriptome sequencing, represents cutting-edge tools for unraveling the intricacies of tissue, organ, cell, and molecular heterogeneity. Looking ahead, with the application of emerging single-cell epigenetics sequencing technologies and rigorous experimental validation, we anticipate a profound dissection of the underlying epigenetic mechanisms governing the interplay between bacteria and tumorigenesis.

## Conclusions

In summary, our study reveals substantial shifts in microbial-derived methyl donor metabolism in CRC, offering a comprehensive understanding of the microbiota-host DNA methylation interplay. Our findings spotlight the pivotal roles of both commensal and pathogenic bacteria, and their methyl donor-related pathways, in modulating DNA methylation to regulate physiological equilibrium and tumorigenesis.

### Supplementary Information

Additional file 1: Fig. S1. Study overview. Fig. S2. The alpha diversity of fecal microbiome derived from CRC patients and healthy controls. Fig. S3. The alpha diversity of tissue microbiome derived from tumor and tumor-adjacent normal tissues. Fig. S4. DNA Methylaiton and taxa-methylation associations in TCGA-CRC cohort. Fig. S5. The differential gene expression of EXO1 between normal and CRC samples. Fig. S6. Interactions between microbes and noncoding DNA methylation. Fig. S7. The gene expression of DNMTs between tumor and normal tissues across TCGA cohort. Fig. S8. The gene expression of TETs between tumor and normal tissues across TCGA cohort.

Additional file 2: Table S1. Information for samples subjected to multiple sequencing. Table S2. Public metagenomic data used for meta-analysis. Table S3. Public 16s rRNA sequencing data used for meta-analysis. Table S4. Differential species between fecal microbiome from CRC patients and healthy controls. Table S5. Differential bacterial pathways between fecal microbiome from CRC patients and healthy controls. Table S6. Differential genus and species between tumor and AN tissues of CRC patients. Table S7. Differential bacterial pathways between tumor and AN tissues of CRC patients. Table S8. Differential EC functions between tumor and AN tissues of CRC patients. Table S9. The association between bacteria abundance between fecal and tissue samples. Table S10. The predicted pathway related to methione and folate metabolism for species associated with DNA methylation. Table S11. The association between host gene methylation and pathways of fecal microbiome.

## Data Availability

All 16S rRNA and metagenomics raw sequencing data have been deposited into CNGB Sequence Archive (CNSA) of China National GeneBank DataBase (CNGBdb) with accession number CNP0004686 (https://db.cngb.org/search/project/CNP0004686/) [71] and CNP0004687 (https://db.cngb.org/search/project/CNP0004687/) [72]. The source code and combined data that was used in the current study are available on GitHub (https://github.com/bioinfolz/CRC-MM) [73].
