# Supplementary material for: Colorectal cancer microbiome programs DNA methylation of host cells by affecting methyl donor metabolism
Source: Genome Med. 2024 Jun 5;16:77. doi: 10.1186/s13073-024-01344-1 (PMC11151592; doi:10.1186/s13073-024-01344-1)
Supplement: Supplementary file 1 — Additional file 1: Fig. S1. Study overview. Fig. S2. The alpha diversity of fecal microbiome derived from CRC patients and healthy controls. Fig. S3. The alpha diversity of tissue microbiome derived from tumor and tumor-adjacent normal tissues. Fig. S4. DNA Methylaiton and taxa-methylation associations in TCGA-CRC cohort. Fig. S5. The differential gene expression of EXO1 between normal and CRC samples. Fig. S6. Interactions between microbes and noncoding DNA methylation. Fig. S7. The gene expression of DNMTs between tumor and normal tissues across TCGA cohort. Fig. S8. The gene expression of TETs between tumor and normal tissues across TCGA cohort. [file 13073_2024_1344_MOESM1_ESM.pdf]

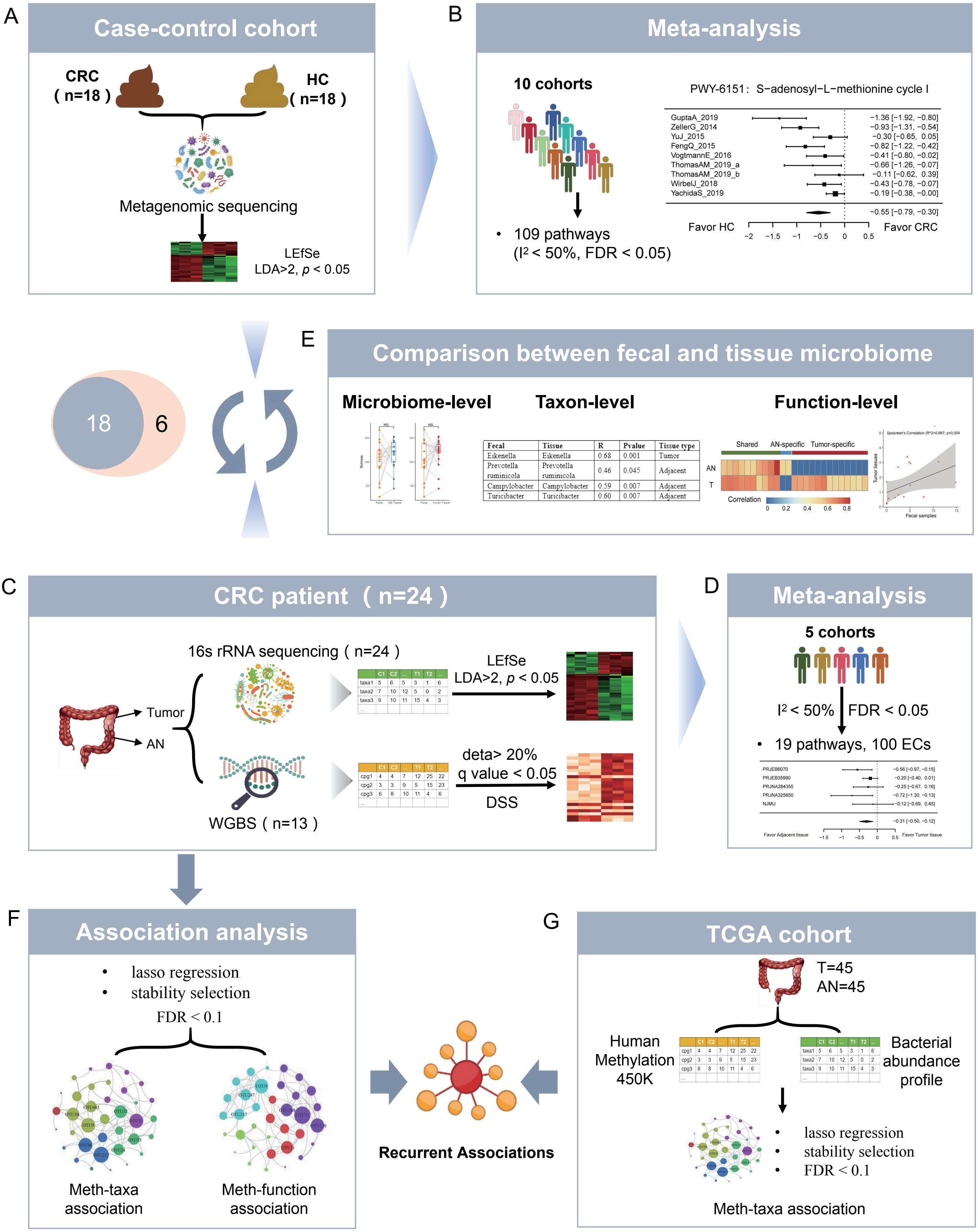

Fig. S1. Study overview. (A) Metagenomic sequencing of fecal samples. (B) Meta-analysis for fecal microbiomes of CRC and healthy controls. (C) 16s rRNA sequencing and WGBS sequencing of tumor and matched tumor adjacent (AN) tissues. (D) Meta-analysis for tissue microbiomes of tumor and AN tissues. (E) Comparison between fecal and tissue microbiome at different levels. (F-G) Methylation-microbes association analysis of our cohort (F) and TCGA CRC cohort (G).

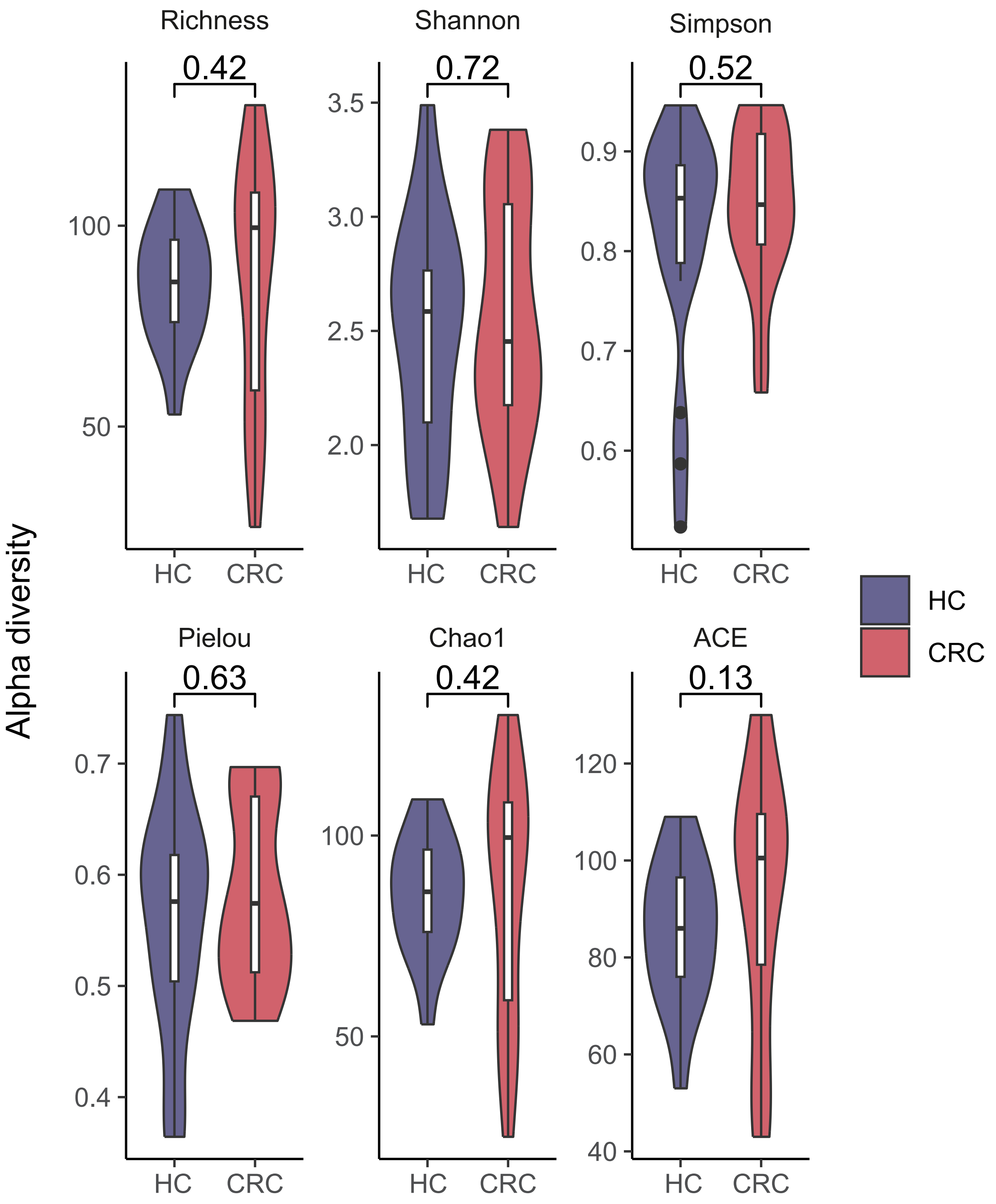

Fig. S2. The alpha diversity of fecal microbiome derived from CRC patients and healthy controls.

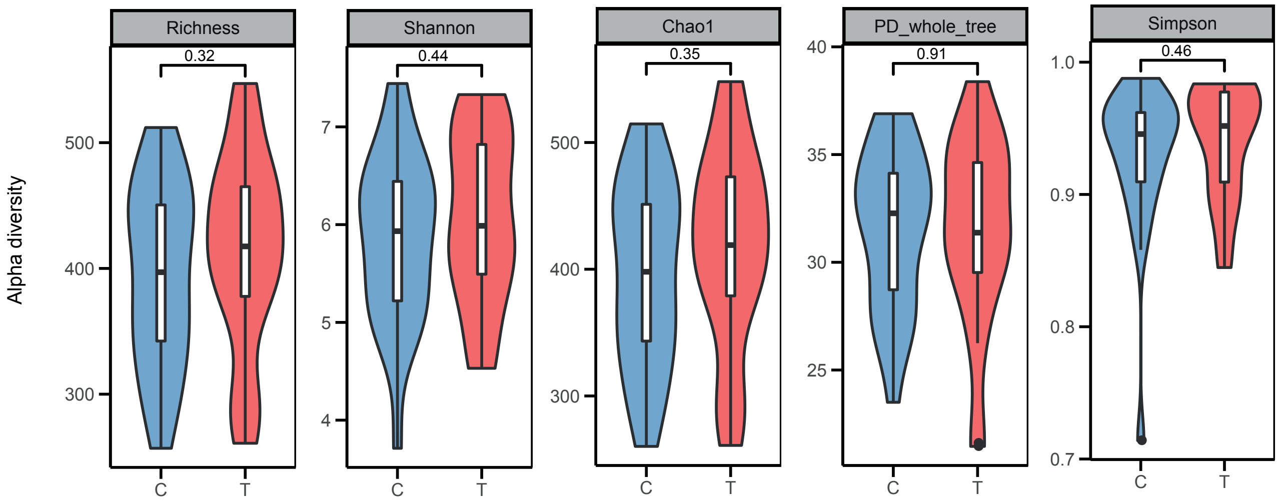

Fig. S3. The alpha diversity of tissue microbiome derived from tumor and tumor-adjacent normal tissues.

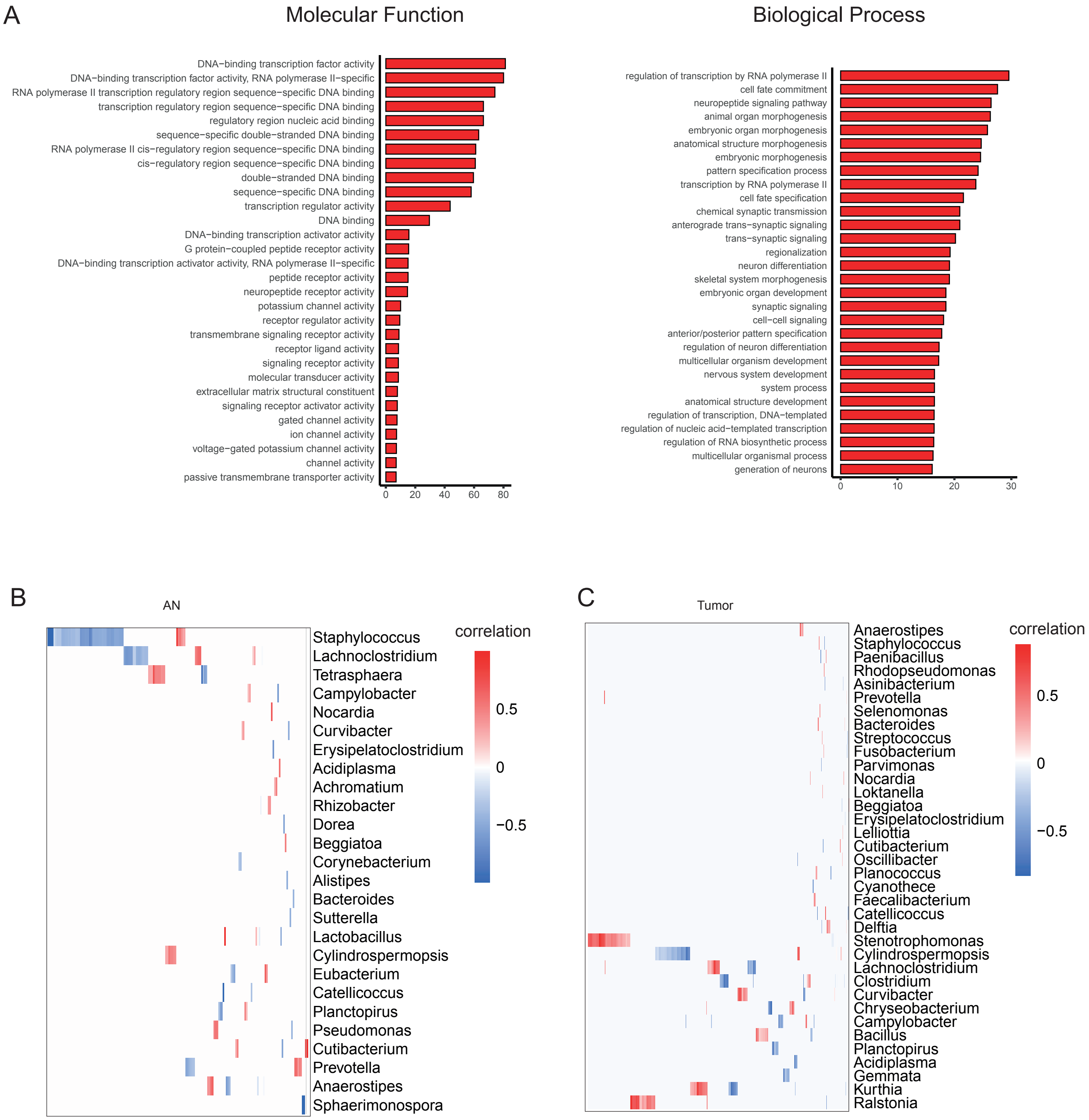

Fig. S4. DNA Methylaiton and taxa-methylation associations in TCGA-CRC cohort. (A). Functional enrichment analysis of differentially methylated genes in TCGA-CRC cohort. (B-C). Heatmap of interaction coefficients between bacteria abundance and promoter methylation in AN (B) and tumor (C) tissues of TCGA-CRC cohort.

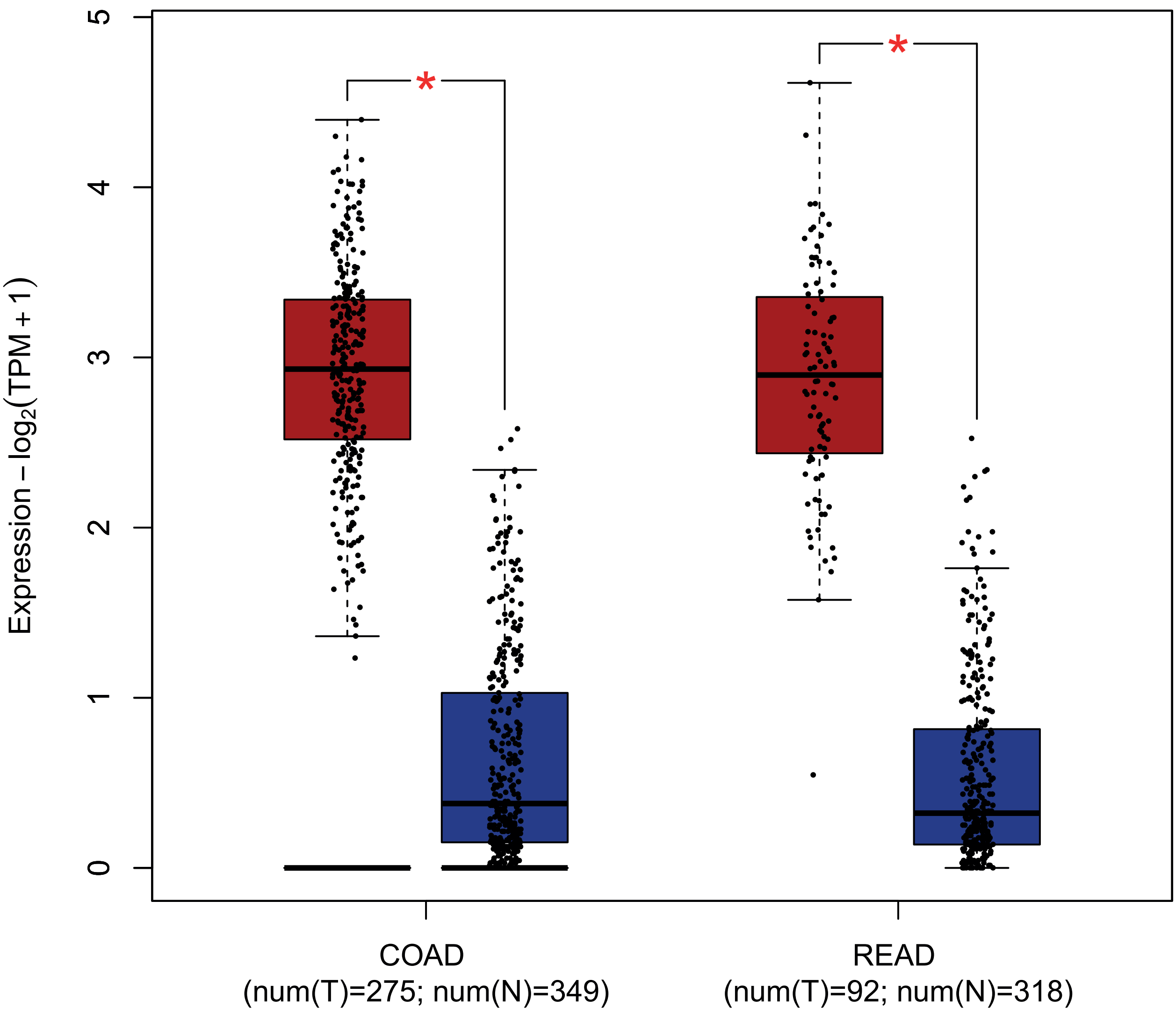

Fig. S5. The differential gene expression of EXO1 between normal and CRC samples. (* *P*<0.05).

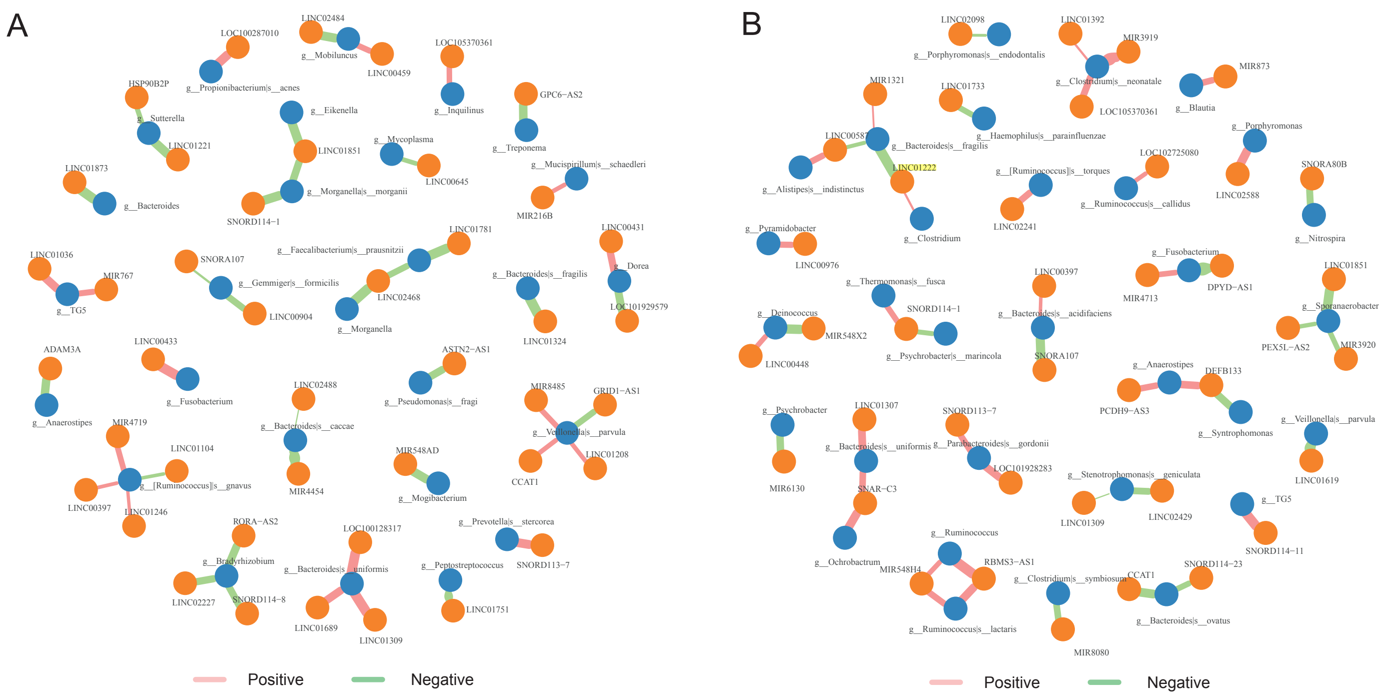

Fig. S6. Interactions between microbes and noncoding DNA methylation. (A-B). Associations between noncoding RNA promoter methylation and intra-tissue microbes in AN (A) and tumor (B) tissues.

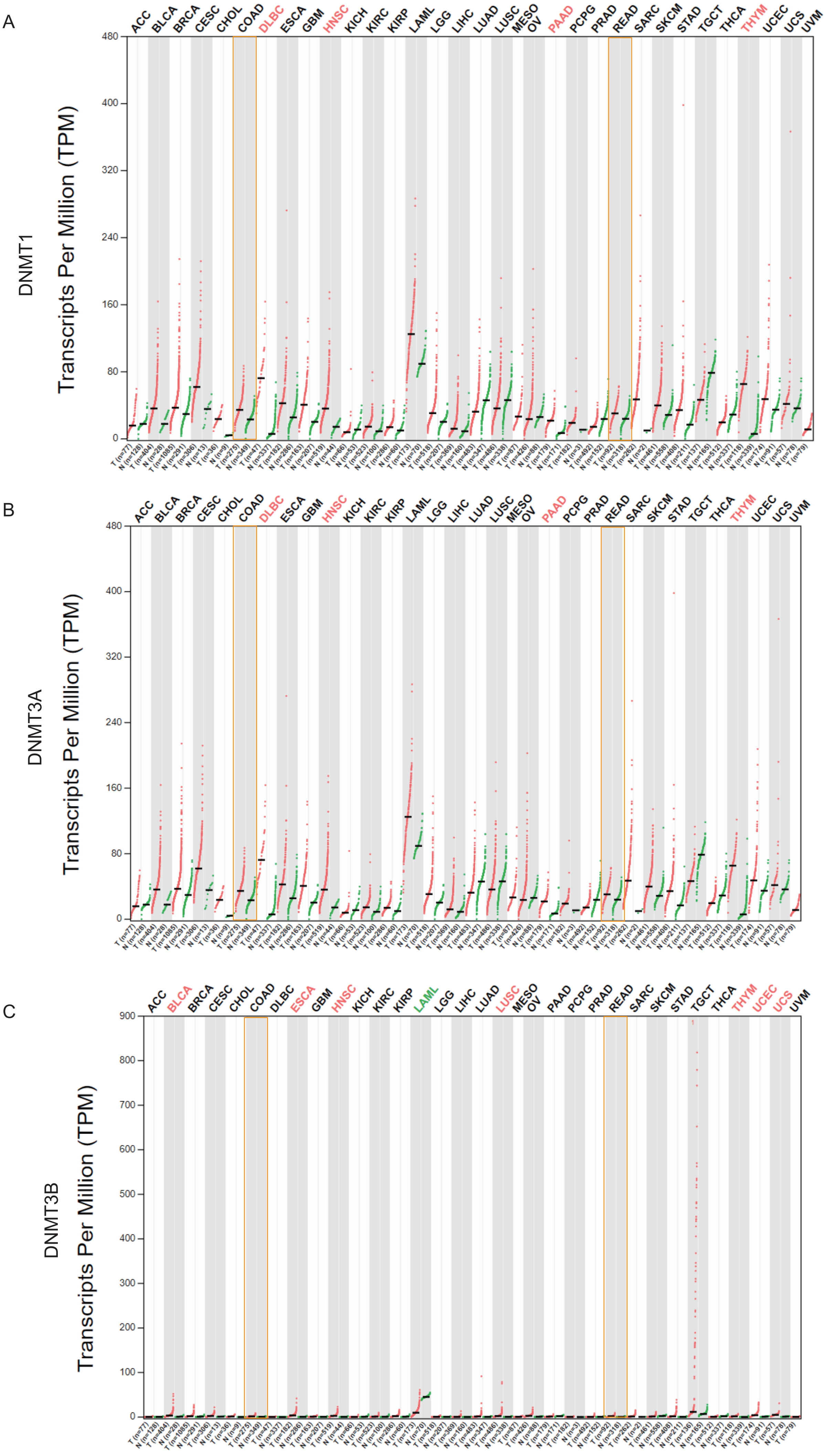

Fig. S7. The gene expression of DNMTs between tumor and normal tissues across TCGA cohort.

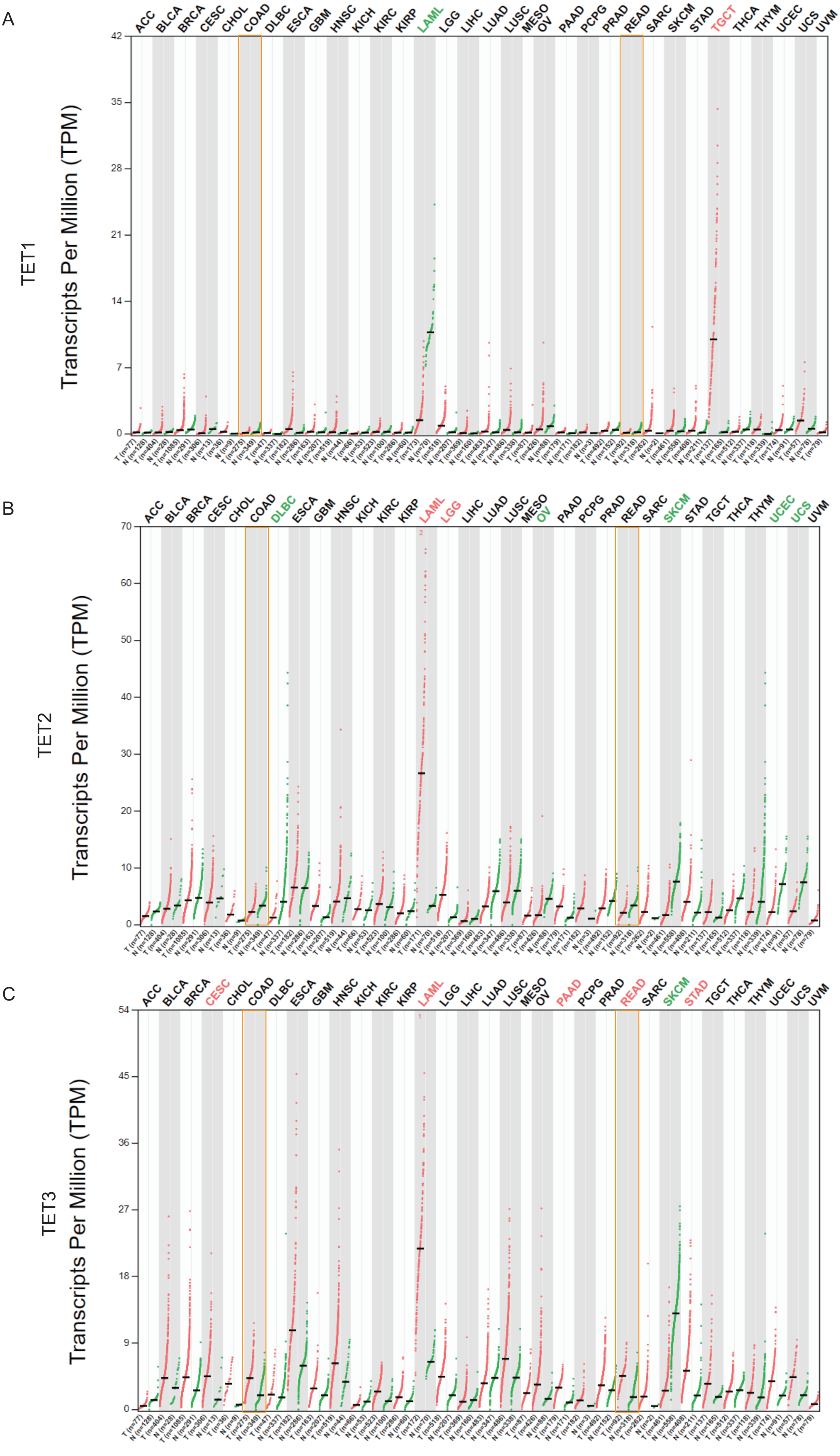

Fig.S8. The gene expression of TETs between tumor and normal tissues across TCGA cohort.
